# Implementation of Nigeria's national surgical plan: building basic life support capacity to strengthen safe surgical care

**DOI:** 10.3389/fmed.2025.1644586

**Published:** 2025-11-12

**Authors:** Aderonke O. Obisesan, Justina O. Seyi-Olajide, Nkeiruka (Nk) Obi, Emmanuel A. Ameh

**Affiliations:** 1 Department of Anaesthesia, National Hospital, Abuja, Nigeria; 2 Department of Surgery, Lagos University Teaching Hospital, Lagos, Nigeria; 3 Smile Trian, Lagos, Nigeria; 4 Department of Surgery, National Hospital, Abuja, Nigeria

**Keywords:** basic life support, national surgical plan, training, emergency preparedness, surgical safety

## Abstract

**Background:**

In many low- and middle-income countries (LMICs), including Nigeria, the burden of surgical conditions significantly contributes to morbidity and mortality rates. Nigeria developed the National Surgical, Obstetrics, Anesthesia, and Nursing Plan (NSOANP) to address gaps in surgical care delivery and improve health outcomes. The success of this plan depends on the preparedness and capacity of healthcare workers, particularly in emergency scenarios. Basic Life Support (BLS) training is essential to improving emergency preparedness and health outcomes.

**Methods:**

This is a retrospective review of 220 participants undergoing a BLS training programme deployed as part of implementation of Nigeria's NSOANP. Trainings were conducted at six locations representing Nigeria's geopolitical zones. Healthcare professionals, including nurses and physicians from specialized cleft care centers and public tertiary hospitals, participated. Post-training evaluations included multiple-choice and skills tests. A qualitative feedback survey assessed participants' self-perception of their skills.

**Results:**

The participants included 151 males (68.6%) and 69 females (31.4%). Most participants were specialist physicians (48.2%) and physician trainees (37.7%). All 220 participants passed the post-test written and skills assessments. Feedback indicated improved understanding of team dynamics and high-quality cardiopulmonary resuscitation. Key recommendations included providing manual defibrillators, incorporating pre-tests, retaining provider manuals, integrating BLS training into postgraduate curricula, and expanding training sessions.

**Conclusion:**

Integrating BLS training into the NSOANP implementation will improve emergency preparedness and competence among healthcare providers. This would potentially improve surgical outcomes. Participants' recommendations offer a roadmap for future improvements. Continued investment in BLS training is essential for building a responsive emergency healthcare system in Nigeria.

## Background

In many low- and middle-income countries (LMICs), including Nigeria, the burden of surgical conditions is substantial, contributing significantly to morbidity and mortality rates (1–3). The World Health Organization (WHO) estimates that 28%−32% of the global burden of disease can be attributed to conditions that require surgical care (1).

In 2015, the Lancet Commission on Global Surgery published its highly impactful work and laid out a roadmap for strengthening surgical care especially in underserved regions (2). The World Health Assembly also made the resolution 68.15 which mandated countries to strengthen surgical care as part of Universal Health Coverage. Following up on these, Nigeria developed her National Surgical, Obstetrics, Anesthesia, and Nursing Plan (NSOANP) to address the gaps in surgical care delivery and improve health outcomes (4, 5). This unique policy document, framed around the core domains of surgical system strengthening has provided a comprehensive framework for enhancing the quality and accessibility of surgical care across the country (2).

As part of the key actions to strengthen workforce capacity building, training in Basic Life Support (BLS) was emphasized in Nigeria's NSOANP. While being highly relevant in the perioperative period, BLS provides immediate care in life-threatening situations irrespective of location, bridges the gap before advanced care and is widely applicable for first responders and healthcare workers (6, 7).

BLS training encompasses a range of essential skills, including cardiopulmonary resuscitation (CPR), airway management, and the use of automated external defibrillators (AEDs) (4). These skills are vital for the initial management of patients experiencing cardiac arrest, respiratory distress, or other life-threatening conditions. Studies have shown that timely and effective BLS interventions can significantly increase the chances of survival and reduce the risk of complications (8). By integrating BLS training into the NSOANP's implementation, Nigeria is operationalizing the “Service Delivery” and “Workforce” domains of its national surgical plan. This approach illustrates how targeted training initiatives can contribute to health system preparedness and the broader goals of health security and resilience, especially in resource-limited settings. This ensures that emergency readiness is not a peripheral activity but a core component of national health planning and policy implementation.

The implementation of Basic Life Support (BLS) training within the framework of Nigeria's National Surgical, Obstetric, Anesthesia, and Nursing Plan (NSOANP) aligns with the global mandate of the World Health Assembly Resolution WHA 76.2, which calls on member states to strengthen emergency, critical, and operative care as part of universal health coverage (UHC) and health systems resilience (9). WHA 76.2 explicitly emphasizes the need to improve the availability and quality of timely emergency interventions—including cardiopulmonary resuscitation—at all levels of the health system, particularly in low- and middle-income countries.

Although the inclusion in surgical plan implementation was done before the WHA resolution 76.2, the creation of the resolution validated the decision to strengthen emergency care under the auspices of surgical care.

In Nigeria, reports have indicated that the knowledge of BLS amongst medical and dental students and physicians is poor (10, 11). In addition, one survey of cardiopulmonary resuscitation capacity at 17 hospitals showed that only 27% had at least one-half of their physicians trained in BLS (9). Against this background, the NSOANP prioritized emergency preparedness and life support within the plan to equip healthcare providers with the necessary skills to manage emergencies, stabilize patients, and improve survival rates in both surgical and non-surgical settings. This is a report of recent experience with implementing BLS training with Nigeria's NSOANP, and it's intended to highlight the importance, feasibility and potential benefits of integrating life support training into surgical plans.

## Materials and methods

This is a retrospective review of the BLS training programme deployed as part of Nigeria's NSOANP implementation conducted from May 2021–April 2023.

### Training locations

The trainings were conducted at 6 different locations, representing the 6 geopolitical zones in Nigeria including:

Kano: North West Zone

Gombe: North East Zone

Abuja: North Central Zone

Lagos: South West Zone

Enugu: South East Zone

Port Harcourt: South South Zone.

### Participant selection

The target participants were healthcare professionals including nurses, trainee physicians and specialist physicians. Participants were selected from specialized cleft lip and palate treatment centers and public tertiary hospitals. The selection was based on their involvement in cleft care and surgical care. The cleft care centers were included as part of a strategy of aligning with funder priorities to achieve broader health system strengthening goals and contributed only 3.6% of the participants. The public tertiary hospitals are multispecialty and offer cleft care as a component of the many specialized services they provide thereby contributing significantly to the broader emergency and surgical preparedness landscape.

### Training protocol

The training was based on the American Heart Association (AHA) BLS training programme. Two AHA BLS certified instructors conducted the training at each location, with each instructor assigned to 6 participants. The training was a one-day programme.

High fidelity feedback cardiopulmonary resuscitation (CPR) mannikins and trainer automatic external defibrillators (AEDs) were used for the training, with a ratio of 3 participants to one mannikin.

The participants received the BLS student's manual to study at least 2 weeks before the training programme. Overall, 4 provider trainings were conducted, involving 220 participants and these have been analyzed.

### Training evaluation

The training evaluation was based on the structure of the AHA BLS training protocol. No pre-test was done. Following training, a multiple-choice post test and skills tests were administered to the participants. A pass mark of 84% in the post test, and 100% in the skills tests were required to be certified as BLS provider. In addition, a qualitative feedback survey, using a 5-point Likert scale, was administered to the participants to ascertain their self-perception of their skills and the training programme.

### Data analysis

Data was analyzed with the Excel Analyse-it^®^ statistical package. For the purposes of analysis, the 5-point Likert scale (strongly agree, agree, neutral, disagree, strongly disagree) was collapsed to a 3-point scale (agree, neutral, disagree). Descriptive data have been expressed as percentages, charts, medians and interquartile ranges.

## Results

### Demographics

There were 151 (68.6%) male and 69 (31.4%) female participants across the 6 geopolitical zones (Table 1).

**Table 1:** Gender and distribution of 220 BLS participants trained in 6 geopolitical zones of Nigeria.

**Geopolitical zones**	**Male (%)**	**Female (%)**	**Total (%)**
North Central	49	34	83 (37.7)
North East	19	2	21 (9.5)
North West	21	2	24 (10.9)
South South	18	5	23 (10.5)
South East	15	7	22 (10.0)
South West	29	19	48 (21.8)
Total	151 (68.6)	69 (31.4)	220 (100)

**Figure 1:**
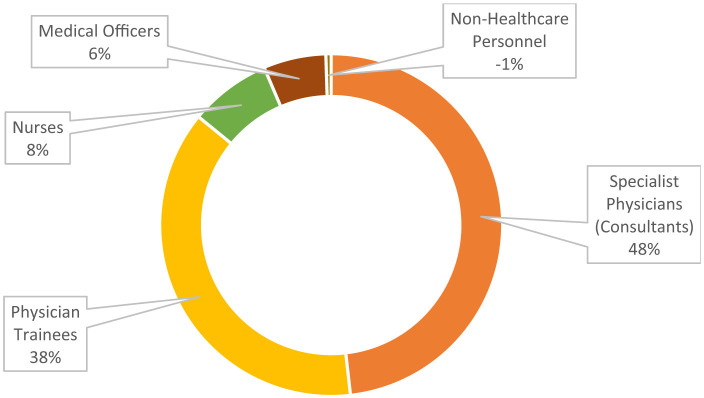
Professional levels of 220 participants undergoing BLS training.

Majority of the participants were specialist physicians (106, 48.2%) and physician trainees (83, 37.7%) (Figure 1). The participants included several physician specialties, nurses and others (Table 2).

**Table 2:** Specialty distribution of 220 participants undergoing BLS training.

**Specialty**	**No. (%)**
Anesthesia	65 (29.5)
Oral and maxillofacial surgery	31 (14.1)
Pediatrics	28 (12.7)
Pediatric surgery	18 (8.2)
Plastic surgery	18 (8.2)
Nursing	17 (7.7)
Medical officer	13 (5.9)
Dentistry	10 (4.5)
Cleft surgeon	8 (3.6)
General surgery	8 (3.6)
Radiology	1 (0.5)
Obstetrics and gynecology	1 (0.5)
Internal/family medicine	1 (0.5)
Non-health care professional	1 (0.5)
Total	220 (100)

### Participants' performance

All 220 participants passed the post test skills and written test and obtained the required pass mark of 84% and were certified in BLS.

### Participants' feedback

**Figure 2:**
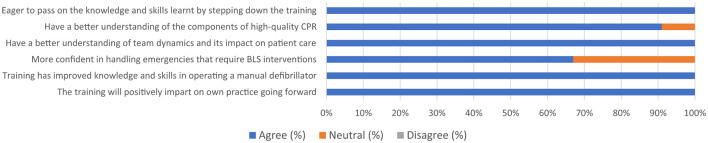
Self-perception of knowledge and skills following training in BLS. BLS, basic life support; CPR, cardiopulmonary resuscitation.

Feedback from the participants showed that all agreed they now had a better understanding of team dynamics and its impact on patient healthcare. Furthermore, all participants agreed they now had a better understanding of the components of high-quality CPR (Figure 2).

Key recommendations from the participants for the future and strengthen life support skills included:

1. To consider donation of manual defibrillators to hospitals where not available in order to enable trained participants to put to practice skills learnt at the training.
2. Pre-test should be included in BLS to better appreciate the improvements with knowledge and skills acquired.
3. The provider manuals should be given to participants for keeps to enable them to refer to it in the course of their practice.
4. This training should be incorporated into the curriculum of postgraduate training in various specialties.
5. More training should be done to expand the volume of providers with expertise in BLS.

### Step down training

One participant has undertaken step down training at her local institution resulting in the training of additional 90 participants. This training though not executed under the AHA programme, has contributed to skills transfer and is a model that is encouraged as a local contextualization of the BLS training focusing on skills acquisition due to high cost of the AHA programme.

## Discussion

One of the key barriers to implementation of BLS education that has been identified is limited government leadership. National level mandates and commitments are needed to address this (12). The integration of Basic Life Support (BLS) training into the National Surgical, Obstetrics, Anesthesia, and Nursing Plan (NSOANP) in Nigeria represents a strategic effort to enhance the preparedness and competence of healthcare providers in managing emergencies. This initiative, with government buy-in addresses a critical need, as surgical conditions contribute significantly to morbidity and mortality rates in low- and middle-income countries (LMICs) like Nigeria (1).

This report demonstrates several key findings that highlight the importance and feasibility of integrating BLS training into surgical plan implementation. The trainings were conducted across six geopolitical zones, ensuring a wide reach, with the potential of addressing regional disparities in healthcare training within the country. Participants included a diverse group of healthcare professionals, primarily specialist physicians and trainee physicians, who are directly involved in surgical and emergency care. This broad participation is crucial for creating a resilient healthcare system capable of responding effectively to emergencies (2).

Although pre-training knowledge was not included in our BLS training programme, others have reported a poor knowledge amongst medical students and physicians in Nigeria (10, 11). In addition, a survey of 553 second to sixth year medical students found that although 79% were aware of BLS, only 29% had knowledge of BLS principles (13). Similarly, a report amongst nurses in Rwanda found baseline knowledge and skills of providing BLS to be poor (14). At institutional level, a survey of 17 hospitals in Nigeria found that only 27% had at least one-half of their physicians trained in BLS (15). These findings highlight the importance and relevance of prioritizing life support training within Nigeria's NSOANP.

The success of the participants at the post test is an indication of the strong immediate knowledge impact of the training. This is similar to other reports showing that knowledge of BLS increased significantly among medical students and nurses after watching a 45-min BLS video and after BLS training respectively (10, 14). However, it has been shown that 6 months after BLS training, only 16% of participants were capable of performing high quality one-rescuer CPR and only 46% scored above 80% (14). This highlights the need for ongoing activities to strengthen knowledge as well as debriefing after every CPR event at institutional levels.

The qualitative feedback from participants reinforced the programme's success. All participants reported a better understanding of team dynamics and high-quality CPR components, essential elements for effective emergency response. The positive feedback highlights the value of hands-on, scenario-based training in improving clinical skills and teamwork in emergency situations (8). However, some participants required remediation to achieve the pass mark, indicating the need for continued practice and reinforcement of skills.

Participants also provided valuable recommendations for future training sessions, emphasizing the need for pre-tests to gauge initial knowledge levels and measure improvements accurately. The suggestion to include manual defibrillators in hospitals reflects a practical step toward ensuring that trained providers can apply their skills in real-life settings. Furthermore, integrating BLS training into postgraduate curricula and expanding the number of training sessions would significantly enhance the sustainability and reach of this initiative (16).

Although the current report has not measured specific metrics related to NSOANP, it's important to emphasize that measuring NSOANP-aligned national indicators, particularly perioperative mortality rate and access to timely emergency care, which serve as critical measures of system performance and patient safety are crucial. By drawing this connection, the value of BLS training should not be assessed solely in terms of the number of participants trained but rather in terms of its contribution to measurable improvements in emergencies and surgical outcomes. In addition, it is planned that future training initiatives will incorporate structured pre- and post-training assessments to capture immediate knowledge and skills acquisition, as well as longitudinal retention tracking at defined intervals to evaluate skill sustainability over time. Linking these individual-level outcomes to broader NSOANP indicators will allow for establishment of a training-to-impact pathway that demonstrates how capacity-building efforts in life support directly strengthen national surgical and emergency preparedness frameworks. This structured evaluation approach ensures that BLS training is not only an educational exercise but also a measurable contributor to Nigeria's progress toward safer surgical systems and improved patient outcomes.

While the initial report of additional participants trained by a locally initiated step-down session is encouraging, its impact remains anecdotal without a standardized assessment approach. To strengthen this model, it's planned that future step-down trainings incorporate formal pre- and post-assessments of knowledge and skills using validated tools to measure immediate learning gains. In addition, longitudinal retention checks would be scheduled at regular intervals to assess how well skills are maintained over time, addressing the well-documented problem of rapid decline in BLS competencies without reinforcement. Beyond individual-level evaluation, institutional-level tracking would be built into the framework to document the application of skills in real-world settings, such as the number and outcomes of resuscitation events, simulation drills, and emergency response audits. Embedding this evaluation structure will ensure that step-down training is not only scalable and cost-effective but also evidence-based, generating measurable data that confirm its effectiveness and its contribution to strengthening Nigeria's emergency preparedness and surgical safety agenda under the NSOANP.

The integration of BLS training into Nigeria's NSOANP aligns with global health strategies that emphasize the importance of emergency and essential surgical care. The Lancet Commission on Global Surgery has highlighted that improving access to surgical and anesthesia care is crucial for achieving universal health coverage (UHC) (2). By equipping healthcare providers with BLS skills, Nigeria can strengthen its emergency care infrastructure, reduce preventable deaths, and improve patient outcomes. For sustainability, there is need for local contextualization using innovative and scalable strategies (17). This is highly encouraged and has already been attempted through the non-AHA based step down training. This model however still requires significant improvement and fine-tuning to ensure standardization.

In conclusion, the implementation of BLS training as part of Nigeria's NSOANP has demonstrated potential benefits in enhancing the emergency preparedness and competence of healthcare providers. The programme's success, evidenced by the positive participant feedback, highlights its potential to improve surgical outcomes. The recommendations provided by participants offer a roadmap for further strengthening and expanding this initiative. Continued investment in BLS training and its integration into broader healthcare plans are essential steps toward building a responsive healthcare system in Nigeria.

Future iterations of the training will go beyond immediate participant numbers and knowledge assessments by integrating evaluation against nationally recognized NSOANP indicators. Specifically, It is planned to link BLS training outcomes with measurable system-level metrics such as surgical volume, perioperative mortality rates, and access to timely emergency care, which are central benchmarks within NSOANP. By aligning programme evaluation with these established indicators, future trainings will not only demonstrate short-term improvements in knowledge and skills but also provide evidence of their contribution to long-term health system strengthening and patient outcomes. This approach ensures that the impact of training can be tracked across individual, institutional, and national levels, thereby creating a more robust training-to-impact pathway and supporting the sustainability and scalability of BLS integration within the broader surgical and emergency care framework.

## Data Availability

The original contributions presented in the study are included in the article/supplementary material, further inquiries can be directed to the corresponding author.
